# Statistical Learning Impairments as a Consequence of Stroke

**DOI:** 10.3389/fnhum.2018.00339

**Published:** 2018-08-28

**Authors:** Albulena Shaqiri, James Danckert, Lauren Burnett, Britt Anderson

**Affiliations:** ^1^Laboratory of Psychophysics, Brain Mind Institute, School of Life Sciences, École Polytechnique Fédérale de Lausanne, Lausanne, Switzerland; ^2^Department of Psychology, University of Waterloo, Waterloo, ON, Canada

**Keywords:** stroke, rehabilitation, statistical learning, neglect, language

## Abstract

Statistical learning is the implicit learning of the contingencies between sequential stimuli, typically from mere exposure. It is present from infancy onward, and plays a role in functions from language learning to selective attention. Despite these observations, there are few data on whether statistical learning capacity changes with age or after brain injury. In order to examine how brain injury affects the ability to learn and update statistical representations, we had young control and healthy elder participants, as well as participants with either left or right brain injury, perform an auditory statistical learning task. Participants listened to two languages with made-up words that were defined by the transition probability between syllables. Following passive listening, learning was assessed with a two-alternative forced choice test for the most familiar word. As in previous studies, we found that young controls have a learning capacity limitation for statistical learning; a second language is less well learned than the first, and this statistical learning capacity limit is attenuated with age. Additionally, we found that brain damaged patients, whether with left or right hemispheric damage, showed impaired statistical learning. This impairment was not explained by aphasia or cognitive deficits. As statistical learning is a critical skill for daily life, a better appreciation of the nature of this impairment will improve our understanding of the cognitive effects of brain injury and could lead to new rehabilitation strategies.

## Introduction

Our ability to quickly learn consistent relationships between sequential stimuli is called statistical learning (Turk-Browne, 2012). One prominent example of statistical learning is our ability to group sounds presented in a consistent order. Present in infancy, the statistical learning of word borders is held to be critical to normal language development (Saffran and Kirkham, 2018). Statistical learning is not, however, restricted to infants and children. This ability persists into adulthood and operates across multiple domains. In the spatial domain, we can learn that some events are more likely to happen in one location than another (Druker and Anderson, 2010; Cort and Anderson, 2013; Jiang et al., 2013). In the temporal domain, we learn that some sequences are more likely than others (Maljkovic and Nakayama, 1996; Fiser and Aslin, 2002), and we learn to predict interval durations (Danckert and Anderson, 2015). Statistical learning does not depend on an active, deliberate search for structure, though it may potentially be aided by such strategies (Gebhart et al., 2009).

Many different brain areas are involved in statistical learning. In a study using a word segmentation task, similar to what is to be reported here, Karuza et al. (2013) found significant changes in metabolic activity in the pars opercularis and pars triangularis of the left frontal lobe. However, when the statistical learning tasks are broadened beyond language based tasks, other brain regions are also highlighted. In their 2015 review, Schapiro and Turk-Browne (2015) reported that the superior temporal gyrus (important for sequential analysis) and the temporal-parietal junction (when regularities are violated) are also frequently found in functional imaging work on statistical learning tasks.

While statistical learning is functionally pervasive, has restricted anatomical correlates, and is present into adulthood, little is known about statistical learning capacity as we grow older, or as a result of brain injury. If statistical learning were to contribute, as seems likely, to implicit learning capacity in elders, then it is likely that events such as strokes would impact it. This would also have an impact on rehabilitation, as current stroke rehabilitation commonly emphasizes learning through repetition.

The hypothesis that brain injury might impair statistical learning after right hemisphere damage is consistent with our prior data on tracking environmental regularities in patients with stroke (Shaqiri and Anderson, 2012; Shaqiri et al., 2013; Stöttinger et al., 2014a,b). However, other data suggests that either hemisphere could be important. Wolford et al. (2000) studied two split-brain participants and a cohort of people with unilateral brain damage. Participants made predictions for two independent sequences of events that could appear in either their right or left visual fields, and thus, be processed by either the left or right hemisphere of the split-brain participants. Depending on which visual field the stimuli were presented in, it was found that both hemispheres were able to form, independently, statistical representations for the stimuli.

Thus, we know that statistical learning is present into adulthood, that it is supported by both hemispheres, and that it is functionally important. What we do not know is how statistical learning is impacted by brain injury, whether there are unique hemispheric effects, nor precisely which brain areas are critical for brain damage to impact statistical learning. We also do not know if statistical learning deficits after brain injury, should such occur, can be re-mediated by massed repetition, breaks, or information about the material to be learned, all of which have been suggested to improve the statistical learning capacity of healthy young individuals (Gebhart et al., 2009; Franco et al., 2011).

We initiated these experiments with several expectations: based on our prior probability learning work (Shaqiri and Anderson, 2013), we expected the presence of neglect to interact with statistical learning deficits. Therefore, we partitioned our right brain damaged (RBD) participants into subgroups with (+N) and without (−N) neglect. In some prior work on updating mental models, we had found differences between left (LBD) and right hemisphere stroke patients (Danckert et al., 2012), and so we also included a LBD group. While many types of learning decline with age, not all do, and this may be particularly true for some forms of implicit memory (Howard and Howard, 2015). Thus, we included both old (OC) and young controls (YC) to compare the stroke patients to participants of the same age, and to address age effects. However, our initial results showed a generally poor performance of all the brain damaged groups. We therefore undertook a more exploratory, less hypothesis driven effort to characterize the extent and nature of the impairment, its sensitivity to manipulations [that had been shown to work in healthy adults (Gebhart et al., 2009)], and a broader investigation of healthy participants to confirm that we were able to reproduce the basic effects reported previously with these testing materials. In the end, we ran several small studies in which all participants were tested on their ability to learn a single language and that allowed us to do a large omnibus test of statistical learning ability. In addition, we could also test the effects of the secondary manipulations in a broad way. Because these secondary manipulations are exploratory, there might be a greater risk of type II error than type I, thus, we have not adjusted for multiple statistical comparisons. Readers should be aware of the exploratory natures of our studies and the risk that any statistical significance may be inflated by multiple comparisons.

The outline of the paper is as follows. We first present the methods for all experiments and for all groups: healthy young, healthy elders, RBD +N, RBD −N, and LBD. We begin the results section with omnibus analyses. We show that our young adult data replicates prior work and that brain damage impairs statistical learning. We report the specific experiments in more detail to look for hints of any beneficial effects of increased exposure, different length rest breaks, or information about the nature of the learning task. Lastly, we discuss the fact that, despite our expectations, damage to either the left or right hemisphere appears equally likely to produce statistical learning impairments. We conclude with an exploratory voxel lesion symptom mapping analysis to ask, regardless of hemisphere, which brain region is most predictive of statistical learning impairments.

## General Methods

### Participants

**FIGURE 1:**
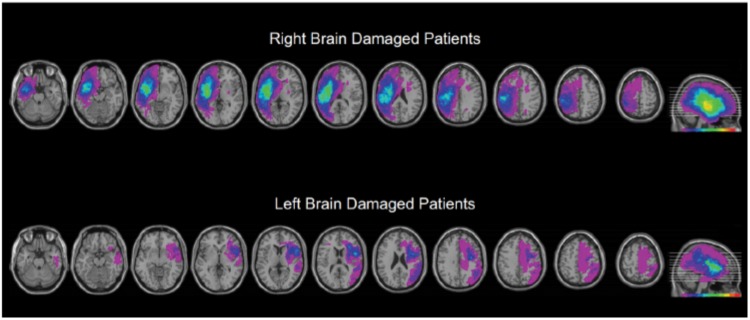
Overview of the extent and prevalence of the lesions in the right and left hemisphere injured groups. As described in more detail in the methods, we manually traced each participant’s brain lesion using the MRIcron software. Individual volumes of interest (VOI’s) were combined into a color coded overlap map. Purple areas indicate single lesions, and the “hotter” the color the greater the number of participants showed overlapping lesions for that region. The index for the heat map is at the bottom of the right most image of each series. The VOIs are mapped to a common brain template with sequential axial slices shown on the left, and a summary sagittal view, indicating the level of the axial slices, shown far right. These data are presented to provide an overview of the extent and location of lesions, and to illustrate that the major location of overlap for the stroke participants was the insular cortex.

This research involved three cohorts: young controls, older controls, and patients with focal brain injury due to strokes (**Table 1**; clinical details on the brain damaged participants are presented as a table in the **Supplementary Materials**). Participants with brain injury generally had middle cerebral artery strokes with variations in size (**Figure 1**). As the testing procedures were largely similar across experiments and participants, we will first describe the cohorts and testing procedures here, and then highlight the slight changes in protocol on an experiment-by-experiment basis. All participants reported English as their principal language. The Office of Research Ethics of the University of Waterloo approved the research and all participants gave written informed consent to participate in the study.

**Table 1:** Stroke patients generally had middle cerebral artery strokes many months prior to participation and with variation in the size and extent.

Participants	Number	Mean age, *SD*	Recruited
Young controls	147 (86 females)	21, 1.98	University of Waterloo
Older controls	23 (9 females)	75, 7.95	Waterloo Research in Aging Participant Pool
Brain damaged – left LBD	15 (6 females)	67, 10.42	Neurological Patients Database in Waterloo
Brain damaged – right RBD-N	17 (9 females)	66, 11.09	Neurological Patients Database in Waterloo
Brain damages- right with neglect RBD+N	9 (5 females)	69, 11.67	Neurological Patients Database in Waterloo

*Details on the clinical participants are provided in a Table in the **Supplementary Materials**. Except for two of the left brain damaged participants and three of the RBD+N, all participants were unique for each experiment. For the five participants who were tested more than once, the testing sessions were conducted at least 1 year apart and these participants did not demonstrate better results than other participants in their groups.*

### Clinical Tests

The older controls and stroke patients underwent testing with a short battery of clinical tests. A detailed table of the clinical details of the brain damaged participants is in the **Supplementary Materials**.

#### Behavioral Inattention Test (BIT)

The BIT (Wilson et al., 1987) was used to test for the presence of spatial neglect. Participants performed star cancelation, letter cancelation, line bisection and copying of the shapes. Participants were diagnosed as having neglect if they scored abnormally on two or more of these subtests: more than 5% of deviation from the center of the lines on the line bisection test, more than 10% of letters or stars missing on the contralesional side for the cancelation tests or failing to copy parts of the three shapes.

#### Five-Item Revised Token Test

In order to confirm that our patients were able to understand and follow task instructions, we used the five-item Revised Token Test (McNeil and Prescott, 1978; revised (RTT): Arvedson and McNeil, 1985) to test for auditory processing and comprehension impairment. The five-item version is a shortened version of the RTT and has been shown to be highly reliable and correlated with the original RTT. For the interpretation of the RTT results, we compared the overall results of our participants with the normative data of 90 normal participants reported by McNeil and Prescott (1978). Five of the brain damaged participants (2 LBD, 2 RBD+N, and one RBD-N) did not take the RTT due to fatigue after the statistical learning and cognitive testing.

#### Montreal Cognitive Assessment (MoCA)

All our participants also performed the Montreal Cognitive Assessment (MoCA), which tests for mild cognitive impairments (Nasreddine et al., 2005). RBD-N patients had a mean score of 27.97, RBD+N patients had a mean score of 21.32, LBD patients had a mean score of 20.77 (mainly due to expressive items being left out) and the controls had a mean score of 27.57. The cut-off of mild-cognitive impairment for the MoCa is a score less than 26.

### Lesion Tracing

The hemisphere affected by participants’ strokes was determined from reviews of their medical records and clinical imaging, such as CT scans. For a general characterization of lesion sizes and locations, we converted the participants’ scans to image files, which were then traced on a standard template and stacked for viewing. For Voxel-based Lesion Symptom Mapping (VLSM), we obtained the original dicom format (Bidgood et al., 1997) for the CT scans (available for 19 RBD and 9 LBD participants). The dicom images were converted to the Nifti format (Li et al., 2016) using the dicom2nii tool that is part of Mricron. Lesions were traced by BA using the Mricron for Linux software. Next, the individual CTs and their matched lesion volumes of interest (voi) were re-centered on the anterior commissure using the SPM8 software (Ashburner et al., 2012). Scans were also reoriented to remove slight tilts, rotations, and yaw on the clinical images. Next, the centered scan and lesion images were converted to the normalized CT scan template using the SPM8 Clinical toolbox plugin (Rorden et al., 2012). VLSM statistical maps were computed using the NPM tool. The Brunner-Munzel statistic was computed and compared to 1000 permutations (with analysis restricted to those voxels affected by lesions for at least one participant; Medina et al., 2010). The lesions of the LBD participants were fewer and more scattered than the RBD participants, with insufficient overlap to support a separate VLSM analysis for the LBD subgroup, but they were included in an omnibus analysis (after “flipping” the hemispheres; reported below). Two of the RBD participants only had CT scans from a time when there was significant coexistent edema, and these two participants were not included in the VLSM analysis. Qualitatively, the VLSM maps for the combined LBD/RBD and RBD only maps were similar and the map of the combined cohort is reported.

### Statistical Learning Testing

Two synthetic languages, denoted A and B, were used in these experiments. Each language was formed by 16 tri-syllabic nonsense words, formed by a combination of six possible vowels (a, ae, e, i, o, u) and six consonants (b, d, k, p, s, t). The latter was variant between the words, but the vowels remained constant (for example, language B was formed with the words “bupaegi,” “tupaeki,” “tedoka,” or “bedoga,” where the vowels u, ae, i and e, o, a, were constant but the consonants changed, see **Table 2**). Language A and B were made by syllables that overlapped by 50%.

**Table 2:** Words for the two test languages with their consonant and vowel frames.

Language A 16 words		Language B 16 words	
Consonants: D, K, B, P, G, T		Consonants: B, P, G, T, D, K	
Vowels frame: A, U, E	Vowels frame: O, I, AE	Vowels frame: U, AE, I	Vowels frame: E, O, A
Da Ku Be	Do Ki Bae	Bu Pae Gi	Be Po Ga
Da Gu Be	Do Gi Bae	Bu Dae Gi	Be Do Ga
Da Ku Te	Do Ki Tae	Bu Bae Ki	Be Po Ka
Da Gu Te	Do Gi Tae	Bu Dae Ki	Be Do Ka
Pa Ku Be	Po Ki Bae	Tu Pae Gi	Te Po Ga
Pa Ku Te	Po Ki Tae	Tu Pae Ki	Te Po Ka
Pa Gu Be	Bo Gi Bae	Tu Dae Gi	Te Do Ga
Pa Gu Te	Po Ge Tae	Tu Dae Ki	Te Do Ka

**FIGURE 2:**
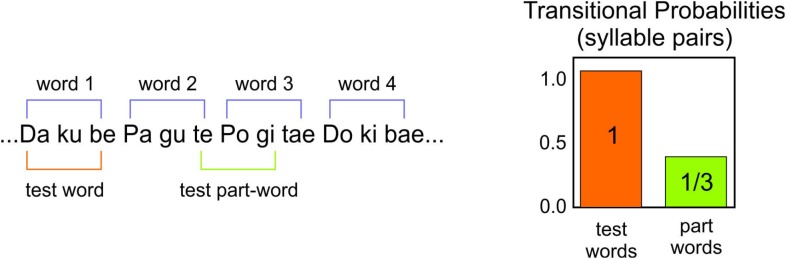
Example of the test words and test part-words of the synthetic languages.

The stimuli were presented using an mp3 player and two headsets. An experimenter listened to the stimuli at the same time as the participants. The testing was conducted in a quiet room. Participants were instructed to listen to the two languages. To assess comprehension, participants performed a two alternative forced-choice task (2AFC) at the end of the listening. Each test item had one test word and one part word. The part words were made up of the syllables 2–3–1 or 3–1–2 that spanned a word border (**Figure 2**). There were 16 items in the test for each language. Participants heard pseudo-randomly either the word or part-word first. After each item there was a brief pause, and participants were asked which of the two words sounded more familiar.

### Statistical Methods

Statistical analyses were performed using the R software (version 3.4.4) (R Core Team, 2018). The logistic linear mixed effects models (Bates et al., 2015) used the R package lme4 (1.1–15). Linear mixed effects models are models with linear components. They are “mixed” in that they have both fixed effects and random effects. Fixed effects are effects one expects one would find on repeating the experiment, such as an effect of a language being the first or second in a series. The random effects are those that might vary with repeating the experiment. For example, we do not expect the Participant 1 in a replication to look like Participant 1 in the original version. The differences in the probability of getting an item correct in the 2AFC task is an example of the dependent variable used here. More details about the lme4 package and linear mixed models can be found in Bates et al. (2015).

## Specific Methods

**FIGURE 3:**
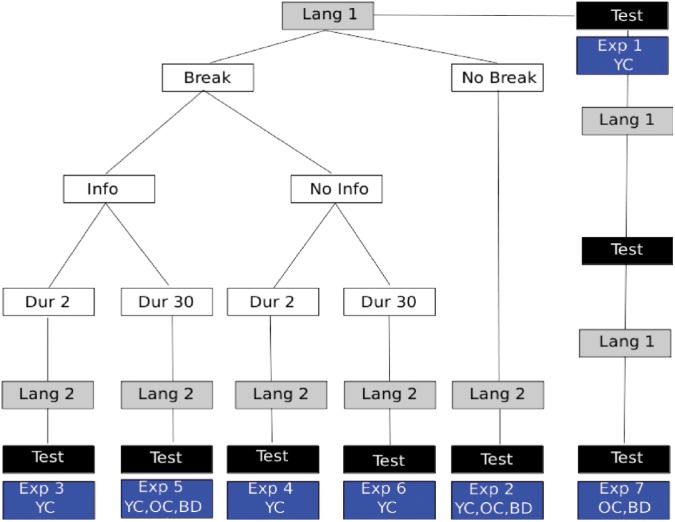
Experiment Overview: language presentation is shown in gray boxes with different conditions described in white boxes. The location of 2AFC assessments for statistical learning is indicated by black boxes. The particular experiment numbers used to indicate the respective manipulations are shown in the blue boxes and described further in the text. Also shown in the blue boxes is which participant groups performed that experimental variant. YC, young controls; OC, healthy elder controls; BD, brain damaged participants, which always included participants with LBD, RBD+N, and RBD–N.

The overview of the different experiments described below is provided in **Figure 3**.

### Experiment 1 – Learning a Single Language

Young control participants listened to one of two pseudo languages for 5 min. The two different versions of the pseudo-language were compared to make sure that the difficulty of learning the two languages was equivalent.

### Experiment 2 – Learning Two Languages – Baseline

Experiment 2 tested for a capacity limit by following the first pseudo-language exposure with a second pseudo-language. After 10 min of total exposure (5 min for each language) participants were given a 32 item forced choice test with 16 items for the first language and 16 items for the second.

### Experiments 3–6 – Effects of Breaks and Information

To test if the statistical learning capacity limitation was modifiable by cues or information, Experiments 3–6 repeated the procedures of Experiment 2 with the following differences. There was either a 2 s break (Experiments 3 and 4) or a 30 s break (Experiments 5 and 6). In addition to the breaks, participants were told (Experiments 3 and 5) or not told (Experiments 4 and 6) what the break signified. See **Figure 3** for a graphical representation of the different experimental conditions. The lengths of the breaks were arbitrary, but were designed to provide either a brief salient signal, or, in addition, a brief moment of respite for possible consolidation. Earlier work had reported that such brief breaks could in fact overcome some of the capacity limit (Gebhart et al., 2009).

### Experiment 7 – Repeated Exposure

To test if massed practice could overcome a statistical language learning impairment, a single 5 min presentation of one language was followed by the 2AFC test (identical to Experiment 1), and this procedure was repeated two more times, resulting in three presentations of the same language and three sequential tests for that same language.

## Results

### Omnibus Test of Statistical Learning

**FIGURE 4:**
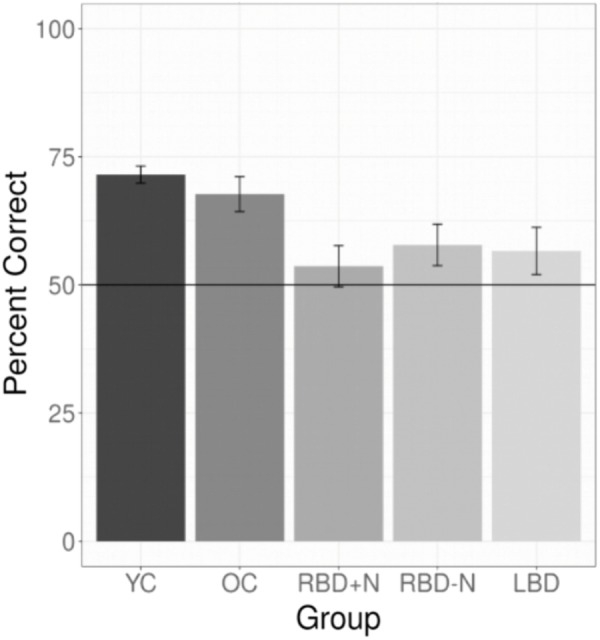
Composite of statistical learning. Collapsing across all experiments and languages for the brain damaged participants and both control groups shows that the brain damaged participants are impaired at statistical learning and that all three brain damaged groups are generally similarly impaired. The whiskers depict standard errors of the mean.

All experiments required participants to learn a language. For experiments 1 through 7 we selected the data for each participant’s first test (E1 and E7) or their test of their first language (all other experiments). This yielded 147 YC, 30 OC, 14 LBD, 16 RBD-N, and 9 RBD+N. The main effect of group was significant with *p*(df = 4) = 0.000 (Chi Square test). The results for the *post hoc* comparisons using Tukey’s method show a general pattern of impairment for the brain damaged participants (**Table 3**). The two control groups are similar to each other and the brain damaged groups are similar to one another as well, and the brain damaged groups do less well than controls. **Table 3** shows the *post hoc* test statistical details. **Figure 4** graphs the groups performances.

**Table 3:** Compares the statistical learning of all brain damaged groups and controls.

Group	*t*-value	*p*-value
YC – RBD+N	−5.10	0.00
YC – RBD−N	−4.50	0.00
YC – LBD	−4.87	0.00
YC – OC	−1.67	0.43
OC – RBD+N	−3.40	0.01
OC – RBD−N	−2.66	0.06
OC – LBD	−2.97	0.02
RBD−N – RBD+N	0.88	0.90
LBD – RBD+N	0.63	0.97
LBD – RBD−N	−0.27	1.00

*For all experiments the data from either the first test (E1 and E7) or the test of the first language was analyzed with a logistic regression model with a group factor. The post hoc Tukey’s test of pairwise comparisons is shown here. Generally, brain damaged groups are worse than controls, and within the controls and brain damaged groups there are only small differences.*

### Omnibus Test of the Effect of a Break and Information

**FIGURE 5:**
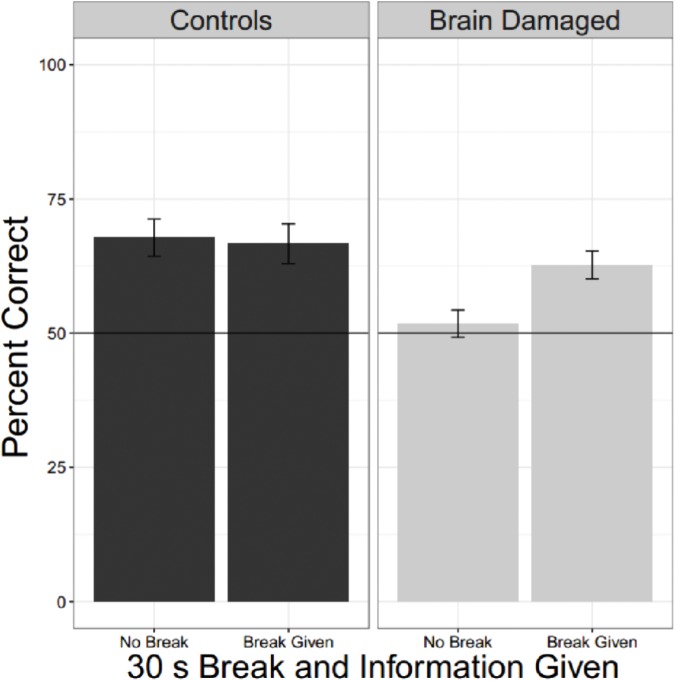
Effect of a break and information. Collapsing across all experiments were brain damaged participants and older controls heard two languages and where there was (or was not) a break reveals that BD are impaired compared to OC, but they also seem to show a tendency to do better when they receive a break and information about the reason for the break.

To compare the effects of a break on second language learning for those with and without brain injury we pooled data from experiments in which participants heard two different languages and each language was, or was not, proceeded by a 30 s break with information. There were 22 unique OC and 29 BD. The linear mixed effects model had fixed factors of BD and whether there was a break as well as a random factor for participant. Results are shown in **Figure 5** and **Table 4**. Brain Damage impairs statistical learning, but there is a suggestion that performance improves with a break and additional information about the task, unlike for OC.

**Table 4:** Comparing the effects of a break for brain damaged and older control participants.

	*z*-value	*P*(>*z*)
Brain damage	−2.48	0.01
Break	1.17	0.24
Interaction	7.68	0.09

### Specific Experiments

#### Experiment 1 – Is There a Difference Between the Two Synthetic Languages Used?

Only young controls were tested in E1 (12 language A and 13 language B). Language learning did occur and was robust. The mean proportion correct was 77.75% (Chi Square = 109.166; *p*-value = 0.000). Performance was not different for the two languages [Two-sample *t*-test (23 df) = 0.87; *p* = 0.4].

#### Experiment 2 – Does Learning One Language Impact Learning a Second?

Young control (*n* = 25) participants learned the first language (77.25%) better than the second (60%; *t* = 3.63; *p*-value = 0.001). Nevertheless, the second language was still learned above chance (Chi Square = 55.208; *p*-value = 0.0003). For the older cohort, there were 10 normal older controls, 5 LBDs, 5 RBDs–N, and 4 RBDs+N.

**FIGURE 6:**
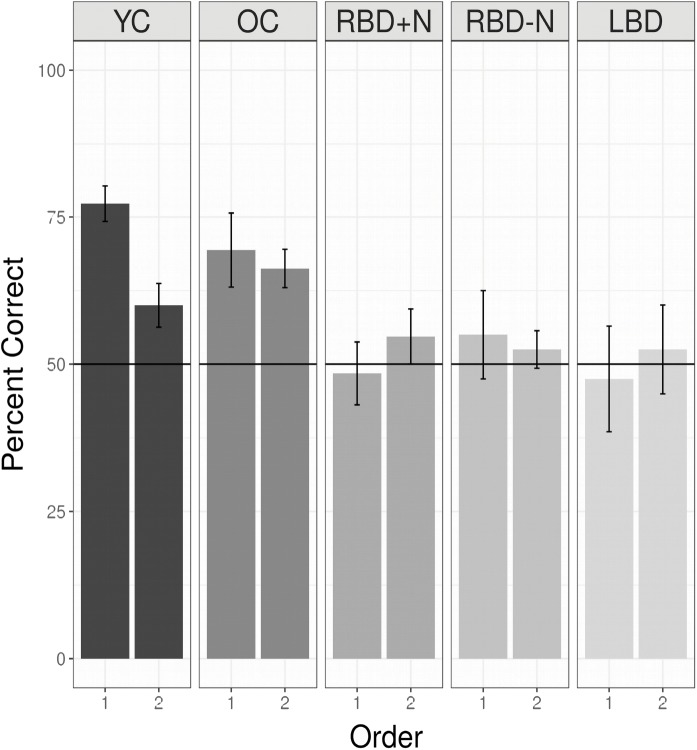
Performance for all groups listening to two languages without a break. Young controls show the nominally highest score on the first language, and a capacity limit. Older controls also demonstrate a statistical learning effect, and are significantly better than all the brain damaged groups, which are at chance levels, and indistinguishable from each other (see above). Chance is indicated by the horizontal line. In addition, OC differ from YC (the reference group) and there is an effect of whether a language is the first or second with this effect reduced for the OC compared to the YC (**Table 5**).

A linear mixed logistic regression model was used to compare young and older controls to the brain damaged groups. A random effect for participant was included. As shown in **Table 5** and **Figure 6** the brain damaged groups perform worse and there is evidence for an attenuated capacity limit for the OC compared to YC.

**Table 5:** Subset of linear mixed logistic regression model coefficients: the baseline is the first language performance of YC.

	*z*-value	Pr(>*z*)
OC	−1.52	0.13
RBD+N	−3.48	0.00
RBD−N	−3.03	0.00
LBD	−3.89	0.00
Order 2	−3.99	0.00
OC × Order 2	2.18	0.03

*Compared to this baseline the brain damaged groups are impaired but the OC are not different. The second language is remembered less well for YC than the first, and there is an interaction where for the OC second language performance is better than the YC second language.*

#### Experiments 2 – 6: Does a Break Effect Statistical Learning Capacity in Healthy Young Adults? And Does it Help if You Are Told There Are Two Languages?

**FIGURE 7:**
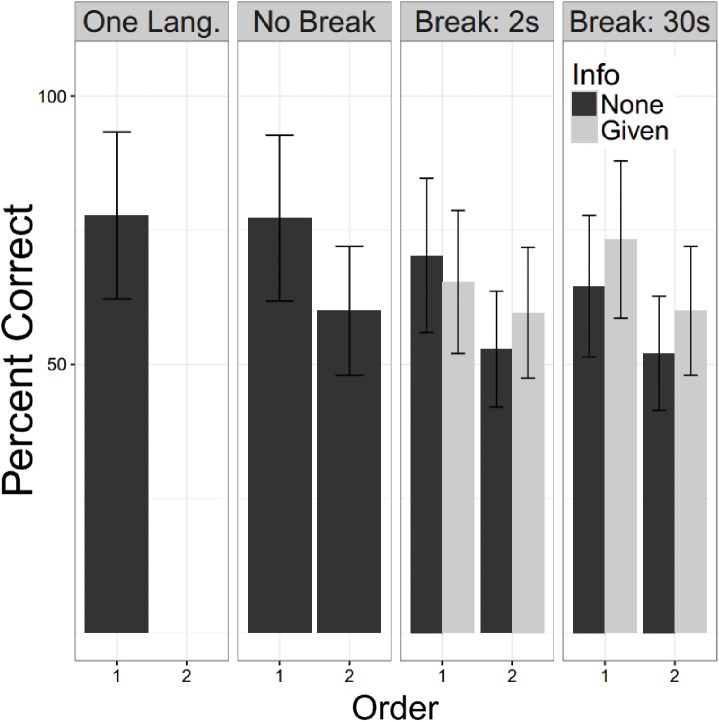
Undergraduate performance. Percent correct is shown for the young controls. The One Lang. Column refers to participants in E1 and No Break for E2. For E3 – 6 the participants are subdivided by whether they were informed of the second language (“Info”). Error bars are plus/minus one standard error from the mean. For all experiments in which two languages were presented, the second language was learned less well than the first. This showed a slight worsening with a break of either duration. We did not confirm the beneficial effect of information reported by Gebhart et al. (2009).

For these analyses all participants heard two language. There were 50 YC with no break and no information (E2), 48 who had a 2 s break with information and 48 who had a 2 S break without information (E3 and E4), and 48 who had a 30 s break with information and 50 a 30 s break without information (E5 and E6). A linear mixed logistic regression model with these participants as a random effect, and fixed effects for the factors of language order, whether a break of 0 s (no break), 2 s or 30 s was given, and, for experiments in which a break was provided, whether or not the participant had been informed of the significance of the break (Info). Testing for fixed effects found that the fixed effects of language order was strongly significant, the second language was recalled less well (*Z* = −5.86; *p* < 0.000). Both durations of breaks were significantly associated with worse recall (2 s: *Z* = −2.65, *p* = 0.008; 30 s: *Z* = −2.49, *p* = 0.0129), while Info did not reach statistical significance, but the trend was for information to improve performance (*Z* = 1.72, *p* = 0.0845). Repeating this analysis for the possible interactions showed no trend toward any significant interaction effects. **Figure 7** shows these patterns graphically with the one language exposure condition of Experiment 1 as a reference.

#### Experiment 7 – Patients: One Language Alone: More Chances to Learn

The prior experiments demonstrate that as a group, stroke patients have trouble with even a simple statistical learning task. In those experiments, participants always heard two languages before being tested on either. Maybe their performance would be better if they were given a single language and tested on it without further exposure? This would decrease their listening time, shorten the time from first language exposure to test, and would be more similar to the way that statistical learning has been tested in children. Or perhaps brain damaged patients are simply on average slower to acquire the statistical associations? In this case, practice and more exposure might improve performance. With these two considerations in mind: less complexity, and more practice, we conducted another experiment where participants heard a single language, were tested immediately, and this cycle was repeated two more times. Participants for these studies were first tested with the screening tools described above. Then they were given the one single language, except that after the first cycle of exposure and test, two more identical cycles were performed. There were 8 normal older controls, 5 LBDs, 6 RBDs-N, and 4 RBDs+N tested for this experiment.

**FIGURE 8:**
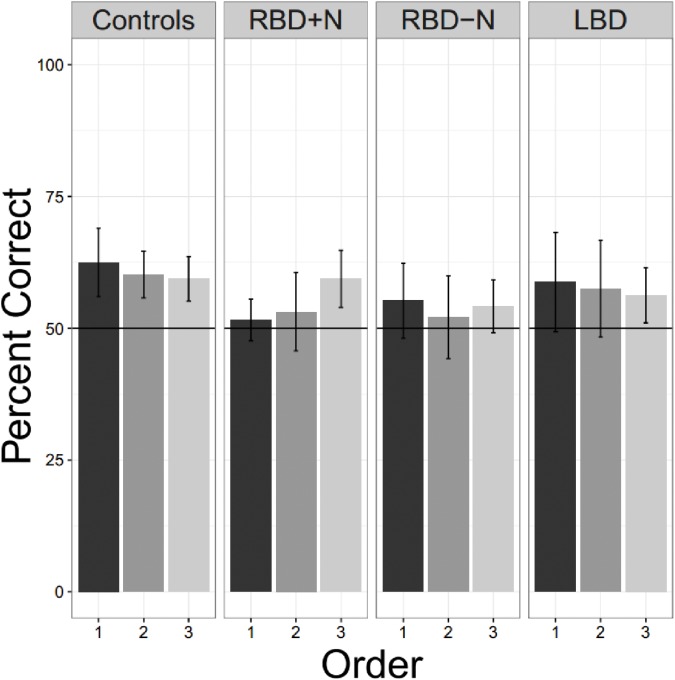
Three exposures to one language. Only the control participants are consistently above chance for all tests. For the RBD+N group, it is clear that 5 min exposure to one language with immediate testing is not sufficient to yield performance above chance.

Even when tested on only a single language, the brain damaged groups did not perform as well as the older controls. Using a test of proportion to see if the groups are significantly above the chance guess rate of 0.5, we found the older controls did learn the first language on the first presentation (Chi Square = 16.00, *p* = 0.025) as did the LBD participants (Chi Square = 11.65, *p* = 0.020). Consistent with the suggestion that the right hemisphere may be more important for statistical learning, the two RBD groups were not statistically significantly different from chance, though the RBD-N group was close (Chi Square = 9.81, *p* = 0.081). There was no hint of learning for the RBD+N (Chi Square = 1.19, *p* = 0.756). Though **Figure 8** might suggest an improvement over time for the RBD+N group, even on the third round the RBD+N group was still not statistically above chance (Chi Square = 2.33, *p* = 0.506).

#### Associations Between Statistical Learning Impairments and Loci of Brain Injury

For comparing lesion location to statistical learning impairments, we used VLSM (Bates et al., 2003) to test for the statistical association between voxel damage and statistical learning as a continuous variable. Although our number of patients for this analysis is relatively small (*N* = 17), our goal, by performing a VLSM, is to explore potential brain areas that might be involved in the brain damaged patients that show statistical learning impairments.

**FIGURE 9:**
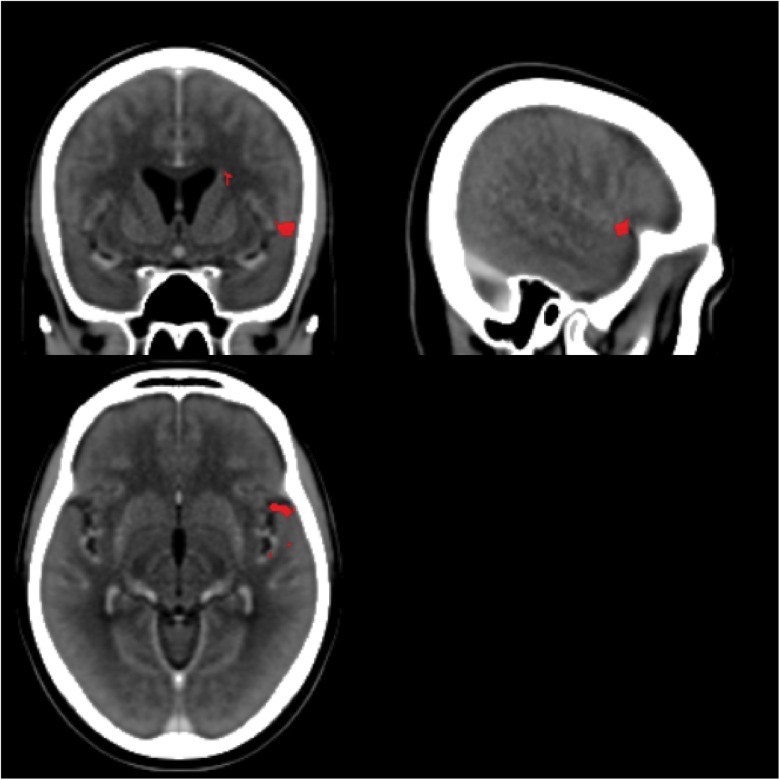
Voxel-based Lesion Symptom Mapping map. The scans for all LBD participants (side of lesion flipped) and RBD participants without significant cerebral edema were combined into a single VLSM analysis. The red areas highlight the area with the greatest statistical association to impaired statistical learning (uncorrected *p*-values spanning 0.02–0.01), but no area exceeded the threshold of a 5% family-wise error. The center of this region is near MNI coordinates 55,11,–6.

Most of our participants had their strokes in the MCA territory with good coverage of this vascular distribution and adequate lesion heterogeneity. Despite this, there was no particular area of the MCA vascular distribution that was statistically associated with an impairment of statistical learning at a family-wise error of less than 0.05 (*z* score 5.2535). The region of the VLSM map with the most extreme *z* statistic was in the anterior area of BA 22 (**Figure 9**; MNI coordinates 55,11,-6 [NB: the leison maps of the patients with left sided lesions had been flipped so the MNI representation on the right side is arbitrary]) near the frontal operculum and anterior insula (**Figure 9**). It is plausible that this result reflects the prevalence with which this territory is damaged by cerebrovascular accidents (Mah et al., 2014). However, it should also be noted that in people without brain damage, nearby and connected areas of this region have been found to be active during tasks that require statistical and perceptual representations (Craig, 2009; Menon and Uddin, 2010; Stöttinger et al., 2014b). Previous studies have also implicated this region in speech production (for a review see Ackermann and Riecker, 2004) with higher activation also evident in this region in bilingual participants. Chee et al. (2004) reported that the insula was more activated when participants spoke two languages with the same proficiency compared to when one language was mastered more than another. The same region has also turned up in functional imaging of people playing the game Rock, Paper, Scissors where changes in functional activity were related to the consequences of prior cycles of the game (Paulus et al., 2005). These data support the conjecture that this peri-insular is involved in functions necessary for learning contingencies or statistical dependencies.

## General Discussion

In the present study, we aimed to test the effects of auditory statistical learning on stroke patients. We found that both right and left hemisphere stroke patients were impaired in learning regularities from an auditory task, and this was true even when breaks or longer exposure times were introduced. Auditory statistical learning is a widely studied paradigm that has been found from infancy to adulthood. Up to now, no study has investigated this function in brain damaged patients. While the principal goal of the research was to examine statistical learning after stroke, we also evaluated young and older controls to provide appropriate baselines, and there are interesting findings for each of these groups as well.

In the young control group, we replicated the robustness of statistical learning, but also the learning capacity limit shown twice before (Gebhart et al., 2009; Franco et al., 2011). The magnitude of this deficit is surprising: with only 5 min exposure to a single statistical stream, the system reaches its limits. While one prior group found that a break and information were sufficient to overcome the deficit (Gebhart et al., 2009), we did not find this. As we used the same testing materials, the different outcomes are not easily explained away as due to procedural approaches.

To our knowledge, we are also the first to examine statistical learning with the classic auditory materials in older people and those with stroke. The only study we have found examining age effects on statistical learning looked at visual statistical learning and used a different paradigm: Campbell et al. (2012) had young and old participants look at visual streams where individual symbols played the part of syllables and sequences of symbols played the part of words. They showed that younger participants were more dependent on attention for statistical learning than the older cohort. It is possible to construe this as a positive effect of age on statistical learning. This interpretation is also confirmed by our results, as we found less of a learning capacity deficit for learning a second language in our older controls.

For the main experimental question, how does stroke affect statistical learning, the answer is: substantially. We found significant effects on the ability to learn a single 5 min pseudo-speech stream in otherwise functional chronic stroke patients. This impairment is not easily accounted for by comprehension deficits or general cognitive impairment. There was no strong evidence of hemispheric lateralization or a particular location that was clearly causal. If a statistical learning impairment is confirmed as a common consequence of brain injury, this will have obvious implications for rehabilitation.

While there are many new and exciting prospects in the field of post-stroke rehabilitation (Brewer et al., 2013), the majority of techniques still rely on practice and repetition (Lohse et al., 2015). Rehabilitation techniques rely on the idea that learning mechanisms remain preserved and will support re-learning. This may be most obvious when applied to motor learning and constraint therapy (Thrane et al., 2015), but it is also clearly required for many occupational and cognitive therapies. Therapy frequently presumes that with practice, the effective actions will come to be the predominant actions. Nevertheless, we show here that learning is impaired in stroke patients. An implication of this finding is that evaluating the effectiveness of a rehabilitation procedure should take into account the preservation (or lack thereof) of statistical learning. For example, rehabilitation methods that rely on implicit learning may not be effective in a patient who has impaired statistical learning abilities. Such methods might be effective though in patients with preserved statistical learning.

We attempted to exclude other reasons for task impairment, such as difficulty in understanding instructions that might masquerade as a statistical learning impairment. We did not find correlations to a measure of general mental status, the MoCA, nor to a measure of the ability to understand and follow instructions (the Revised Token Test). There was also no correlation with the volume of the stroke (data not shown).

## Conclusion

We found evidence of impaired statistical learning following strokes of left and right hemispheres. There was no clear explanation in terms of language deficits or a generalized cognitive impairment. A statistical learning impairment was seen in those with and without spatial neglect. We think those results are highly relevant for post-stroke rehabilitation. Many aspects of rehabilitation are essentially skill learning (Krakauer, 2006; Sigrist et al., 2013). In skill learning, much of the improvement is arguably due to the implicit acquisition of probabilistic contingencies and biases. If those processes are impaired by brain injury, then rehabilitation strategies that rely on them are impacted. Measuring deficits in statistical learning could decrease the unexplained heterogeneity in patient studies and might be helpful when doing research on compensatory approaches.

## Author Contributions

BA and AS designed the experiments. AS collected the data. AS, BA, and LB carried out the analyses. AS, BA, and JD wrote the manuscript.

## Conflict of Interest Statement

The authors declare that the research was conducted in the absence of any commercial or financial relationships that could be construed as a potential conflict of interest.

## Supplementary Material

TABLE S1 Clinical details of brain damaged participants. Columns show the age, sex (F = female, M = Male), time between stroke and testing in months, group assignment (LBD = left brain damage, RBD = right brain damage, RBN+N = right brain damage with neglect), experiments participated in, Lesion = area of damage, F = frontal, P = parietal, T = temporal, O = occipital, RO = rolandic operculum, Ins = insula, Th = thalamus, and BG = basal ganglia. MoCA = the MoCA score (> = 26 is “normal”), Token = Token Test Score, Line = Line Bisection Error, Star = Stars Omitted, Letter = Letter Cancellation Task Score, Copy = qualitative observations of the Copy Task. The last four columns are components of the Behavioral Inattention Test.
