# Supplementary material for: Statistical Learning Impairments as a Consequence of Stroke
Source: Front Hum Neurosci. 2018 Aug 28;12:339. doi: 10.3389/fnhum.2018.00339 (PMC6121198; doi:10.3389/fnhum.2018.00339)
Supplement: TABLE S1 — Clinical details of brain damaged participants. Columns show the age, sex (F = female, M = Male), time between stroke and testing in months, group assignment (LBD = left brain damage, RBD = right brain damage, RBN+N = right brain damage with neglect), experiments participated in, Lesion = area of damage, F = frontal, P = parietal, T = temporal, O = occipital, RO = rolandic operculum, Ins = insula, Th = thalamus, and BG = basal ganglia. MoCA = the MoCA score (> = 26 is “normal”), Token = Token Test Score, Line = Line Bisection Error, Star = Stars Omitted, Letter = Letter Cancellation Task Score, Copy = qualitative observations of the Copy Task. The last four columns are components of the Behavioral Inattention Test. [file Table_1.DOCX]

| Age | Sex | Interval in Months between Stroke and Experimental Participation | Group | Exp | Lesion | MoCA | Token | Line | Star | Letter | Copy |
| --- | --- | --- | --- | --- | --- | --- | --- | --- | --- | --- | --- |
| 69 | M | 73 | LBD | 2, 7 | T, P | 16  18 | 11.71 | 3.95%  0.7% | 3/28, 1/28  0/28, 4/28 | 1/10 6/10 5/10  2/10  0/10  0/10  0/10  1/10 | OK  Cube left bottom missing |
| 71 | F | 13 | LBD | 5 | T, O | 20 | 13.01 | 1.69% | 0/28, 0/28 | 4/10  1/10  3/10  2/10 | Ok |
| 65 | M | 38 | LBD | 5, 7 | F, RO, T, O, Ins | 16 (Aphasia) | 12.86  13.09 | 1.97%  1.27% | Ok  Ok | Ok  Ok | Ok  Ok |
| 60 | F | 278 | LBD | 5 | F, P | 21 | 13.94 | 0.7% | Ok | Ok | Ok |
| 73 | M | 40 | LBD | 5 | F, RO, Ins | 16 (Aphasia) | 12.16 | 1.27% | 1/28, 0/28 | 1/10 3/10 2/10 2/10 | Cube misses top part |
| 60 | M | 24 | LBD | 2 | P | 3 (Severe aphasia) | 11.93 | 3.53% | 4/28, 1/28 | 1/10  3/10  1/10  0/10 | Cube is a square |
| 72 | F | 24 | LBD | 2 | F, RO, T, Ins | 26 | 13.95 | 1.69% | Ok | 2/10  1/10  3/10  1/10 | Ok |
| 65 | M | 95 | LBD | 2 | P, O, T, RO | 21 | 13.23 | 0.56% | Ok | Ok | Ok |
| 66 | F | 28 | LBD | 5 | F, T, RO, Ins | 28 | 14.40 | 2.96% | Ok | Ok | Ok |
| 77 | F | 42 | LBD | 2 | Ins | 27 | 13.7 | 1.97% | 0/28  1/28 | Ok | Ok |
| 68 | M | 36 | LBD | 5 | F, RO, Ins | 29 | 14.5 | 2.68% | Ok | Ok | Ok |
| 88 | M | 23 | LBD | 5 | F, RO, Ins | 8 (Aphasia) | 13.31 | 6.77% | 0/28  11/28 |  | Cube and flower bottom part missing |
| 79 | M | 44 | LBD | 7 | F, P | 30 |  | 0.84% | ok | 0/10  0/10  1/10  0/10 | ok |
| 48 | M | **59** | LBD | 7 | P, T, O, RO | 26 |  | 0.56% | 0/28  1/28 | 1/10  1/10  1/10  0/10 | Cube right side missing |
| 50 | F | 111 | LBD | 7 | F | 29 | 14.65 | 1.55% | 1/28  0/28 | 0/10 0/10 0/10 1/10 | Cube and flower left side missing |
| 66 | F | 2m | RBD | 5 | F, BG, O, P, T, RO, Th, Ins | 29 | 14.56 | 2.4% | 1/28  3/28 | Ok | Ok |
| 67 | F | 13 | RBD | 2 | P | 27 | 14.8 | 1.97% | Ok | Ok | Ok |
| 57 | F | 15 | RBD | 5 | F, P, T, O, RO, Ins | 27 | 14.06 | 1.69% | 2/28  2/28 | 0/10  0/10  1/10  2/10 | Cube left side is distorted |
| 83 | F | 24 | RBD | 2 | F, BG, T, RO, Th, Ins | 29 | 14.21 | 0.56% | 1/28  0/28 | 0/10  0/10  0/10  1/10 | Star left side distorted |
| 70 | F | 32 | RBD | 5 | F, T, RO, P, Ins | 28 | 14.66 | 0.56% | 9/28  5/28 | 0/10  1/10  2/10  2/10 | Ok |
| 72 | F | 30 | RBD | 2 | F, T, RO, Ins | 28 | 14.66 | 1.12% | 0/28  1/28 | 1/10  0/10  1/10  1/10 | Ok |
| 67 | M | 39 | RBD | 2 | F, BG, T, Th, Ins | 30 | 14.44 | 4.5% | 1/28  3/28 | Ok | Ok |
| 67 | M | 1m | RBD | 5 | F, BG, O, P, T, RO, Ins | 28 | 14.8 | 1.27% | ok | 1/10  0/10  2/10  3/10 | Ok |
| 90 | M | 8m | RBD | 2 | O, P | 26 | 14.06 | 0.7% | 4/28  2/28 | 2/10  0/10  2/10  2/10 | Ok |
| 59 | M | 6m | RBD | 5 | F, BG, O, P, T, RO, Th, Ins | 29 | 14.79 | 0.98% | 1/28  0/28 | 1/10  0/10  2/10  0/10 | Ok |
| 75 | F | 111 | RBD | 7 | P, BG | 29 | 14.505 | 2.54% | Ok | 0/10  0/10  1/10  0/10 | Ok |
| 45 | M | 54 | RBD | 7 | P, T, O, RO, Ins | 28 | 14.42 | 0.98% | Ok | 1/10  0/10  1/10  0/10 | Ok |
| 80 | F | 172 | RBD | 7 | P, O | 29 | 14.53 | 5.08% | Ok | Ok | Ok |
| 58 | M | 132 | RBD | 7 | T, BG, RO, Ins | 24 | 14.05 | 1.12% | Ok | 0/10  0/10  1/10  0/10 | Ok |
| 57 | M | 2m | RBD | 7 | T, RO, Ins | 30 | 14.815 | 1.97% | Ok | Ok | Ok |
| 59 | M | 54 | RBD | 7 | P | 23 | 14.53 | 3.81% | 0/28  1/28 | 0/10  0/10  2/10  0/10 | Ok |
| 62 | F | 53 | RBD | 7 | T, RO, Th, Ins | 27 |  | 2.4% | 4/28  2/28 | Ok | Ok |
| 59 | M | 3m | RBD+N | 2 | P, O | 18 | 12.68 | 6.49% | 6/28  4/28 | 7/10  9/10  5/10  5/10 | Star left side distorted. Cube and flower left side missing |
| 84 | F | 3m | RBD+N | 5 | F, T, RO, Ins | 21 | 14.4 | 5.22% | 18/28  0/28 | 9/10  1/10  6/10  2/10 | Cube and flower left side missing |
| 64 | F | 1m | RBD+N | 2 | P, F, T, RO, O, Ins | 22 | 14.07 | 28.53%  15.81% | 28/28  13/28  28/28  2/28 | 10/10  10/10  10/10  0/10  10/10  10/10  1/10  0/10 | Cube and flower left side missing  Cube bottom part missing |
| 66 | F | 24 | RBD+N | 5, 7 | Th | 22 | 13.94 | 9.88%  10.02% | 28/28  13/28  28/28  15/28 | 10/10  10/10  10/10  2/10  10/10  10/10  4/10  0/10 | Star, cube and flower left side missing  Star, cube and flower left side missing |
| 86 | M | 10m | RBD+N | 5 | P, T, O, RO, Ins | 26 | 14.11 | 6.07% | 12/28  8/28 | 1/10  1/10  0/10  2/10 | Star and cube left side missing |
| 71 | M | 2m | RBD+N | 2 | F, T, BG, O, P, RO, Th, Ins | 25 |  | 9.03% | 17/28  0/28 | 3/10  2/10  2/10  3/10 | Cube left side missing |
| 75 | F | 12 | RBD+N | 2, 7 | F, T, BG, P, RO, Ins | 11 | 14.24 | 7.76%  15.96% | 3/28  1/28  27/28  5/28 | 4/10  0/10  3/10  1/10  10/10  10/10  10/10  1/10 | Cube left side missing  Cube left side missing |
| 71 | F | 26 | RBD+N | 5 | F, T | 27 | 14.9 | 4.37% | 8/28  1/28 | 2/10  0/10  0/10  0/10 | Cube left side missing |
| 49 | M | 24 | RBD+N | 7 | F, T, BG, P, RO, Th | 28 |  | 4.23% | 1/28  0/28 | 2/10  2/10  0/10  0/10 | Ok |
